# Technical Performance Evaluation of Olink Proximity Extension Assay for Blood-Based Biomarker Discovery in Longitudinal Studies of Alzheimer's Disease

**DOI:** 10.3389/fneur.2022.889647

**Published:** 2022-06-06

**Authors:** Becky C. Carlyle, Robert R. Kitchen, Zoe Mattingly, Amanda M. Celia, Bianca A. Trombetta, Sudeshna Das, Bradley T. Hyman, Pia Kivisäkk, Steven E. Arnold

**Affiliations:** ^1^Department of Neurology, Massachusetts General Hospital and Harvard Medical School, Boston, MA, United States; ^2^Cardiovascular Research Center, Massachusetts General Hospital and Harvard Medical School, Boston, MA, United States

**Keywords:** biomarker, Alzheimer's disease, plasma, neurodegeneration, biotemporal stability, Olink

## Abstract

The core Alzheimer's disease (AD) cerebrospinal fluid (CSF) biomarkers; amyloid-β (Aß), total tau (t-tau), and phosphorylated tau (p-tau181), are strong indicators of the presence of AD pathology, but do not correlate well with disease progression, and can be difficult to implement in longitudinal studies where repeat biofluid sampling is required. As a result, blood-based biomarkers are increasingly being sought as alternatives. In this study, we aimed to evaluate a promising blood biomarker discovery technology, Olink Proximity Extension Assays for technical reproducibility characteristics in order to highlight the advantages and disadvantages of using this technology in biomarker discovery in AD. We evaluated the performance of five Olink Proteomic multiplex proximity extension assays (PEA) in plasma samples. Three technical control samples included on each plate allowed calculation of technical variability. Biotemporal stability was measured in three sequential annual samples from 54 individuals with and without AD. Coefficients of variation (CVs), analysis of variance (ANOVA), and variance component analyses were used to quantify technical and individual variation over time. We show that overall, Olink assays are technically robust, with the largest experimental variation stemming from biological differences between individuals for most analytes. As a powerful illustration of one of the potential pitfalls of using a multi-plexed technology for discovery, we performed power calculations using the baseline samples to demonstrate the size of study required to overcome the need for multiple test correction with this technology. We show that the power of moderate effect size proteins was strongly reduced, and as a result investigators should strongly consider pooling resources to perform larger studies using this multiplexed technique where possible.

## Introduction

Diagnosis of Alzheimer's disease (AD) and AD-related dementias (ADRD) historically depended on clinical features (“typical” symptoms, signs and course) and exclusion of other potential causes of cognitive decline, with identification of abundant plaque, tangle or other histopathologies such as lewy bodies and TDP-43 inclusions at autopsy being the gold standard method for definitive diagnosis. With the development of amyloid-β and tau imaging and biofluid biomarkers, we can now come very close to definitive diagnosis during life. However, our understanding of the pathology and pathophysiology of AD, beyond the presence or absence of amyloid-β and tau, requires identification and characterization of a wide range of biological factors that drive neurodegeneration over time and different stages of disease. Repeat biofluid sampling to measure biomarker changes over time is essential for further disease characterization, staging, monitoring progression, and as secondary outcomes in clinical trials (1, 2). Lumbar puncture for cerebrospinal fluid (CSF) biomarkers in AD is useful to diagnose individuals in research studies, but has not been widely adopted, at least in part because of acceptability and challenges for repeat sampling (3–5). Thus, there is immense demand for blood-based biomarkers in research studies as well as clinical practice as plasma collection is easy and practical, allowing more frequent measurements.

AD/ADRDs pose some unique challenges to the biomarker discovery process given significant pathophysiological heterogeneity within and between diseases and overlapping comorbidities with systemic metabolic, cardiovascular, and inflammatory diseases (6–8). The pathophysiological drivers and associations of these comorbidities with neurodegeneration may also change over time. Understanding the longitudinal stability (intra-individual variation in a biological analyte over time, or biotemporal stability) is a requisite for establishing a candidate biomarker's utility in clinical and research settings (9). Repeat samplings over time inform analyte fluctuations relative to an individual baseline, useful for evaluating therapeutic effects of interventions in clinical trials or disease progression monitoring (3, 10, 11). It is critical that the tools for biomarker characterization are precise and reproducible to meet these demands.

In order to explore the feasibility of this technology for discovering blood-based biomarkers across all fields of medicine, but with a focus on Alzheimer's Disease, we independently evaluated the technical performance of Olink Proteomics high-throughput, multiplex proximity extension assays (PEA) for protein screening (12, 13). Five commercial protein panels were chosen for measurement precision and reproducibility analysis. Our control structure included placing three plasma samples, which were independent from the Olink control samples, to be run in duplicate on every plate for analysis of technical precision, and multiple annual samples from the same subjects for analysis of biotemporal stability. For the majority of analytes measured, Olink's PEA technology performed well, exceeding our standard performance criteria while requiring small sample volume. A high degree of intra- (within plate) and inter- (between plate) measurement precision demonstrated technical robustness and performance reliability, supporting its utility in simultaneous evaluation of many analytes. Biotemporal variability is often unheeded in biomarker studies. Our evaluation found the majority of analytes to be relatively stable over time, with the preponderance of inter-individual analyte variability attributed to diagnosis and biological variations between individuals. Proteins with large within group variability or with small to medium differences between diagnostic groups are particularly impacted by stringent statistical significance thresholds in multi-protein studies. For this reason, a power calculation was performed to address sample size requirements, as the need to correct for multiple tests is a notable limitation of using this technology. Overall, the technical robustness and reliable performance of this technology supports its utility for simultaneous evaluation of many blood-based candidate biomarkers.

## Methods

### Experimental Cohort

**Figure 1:**
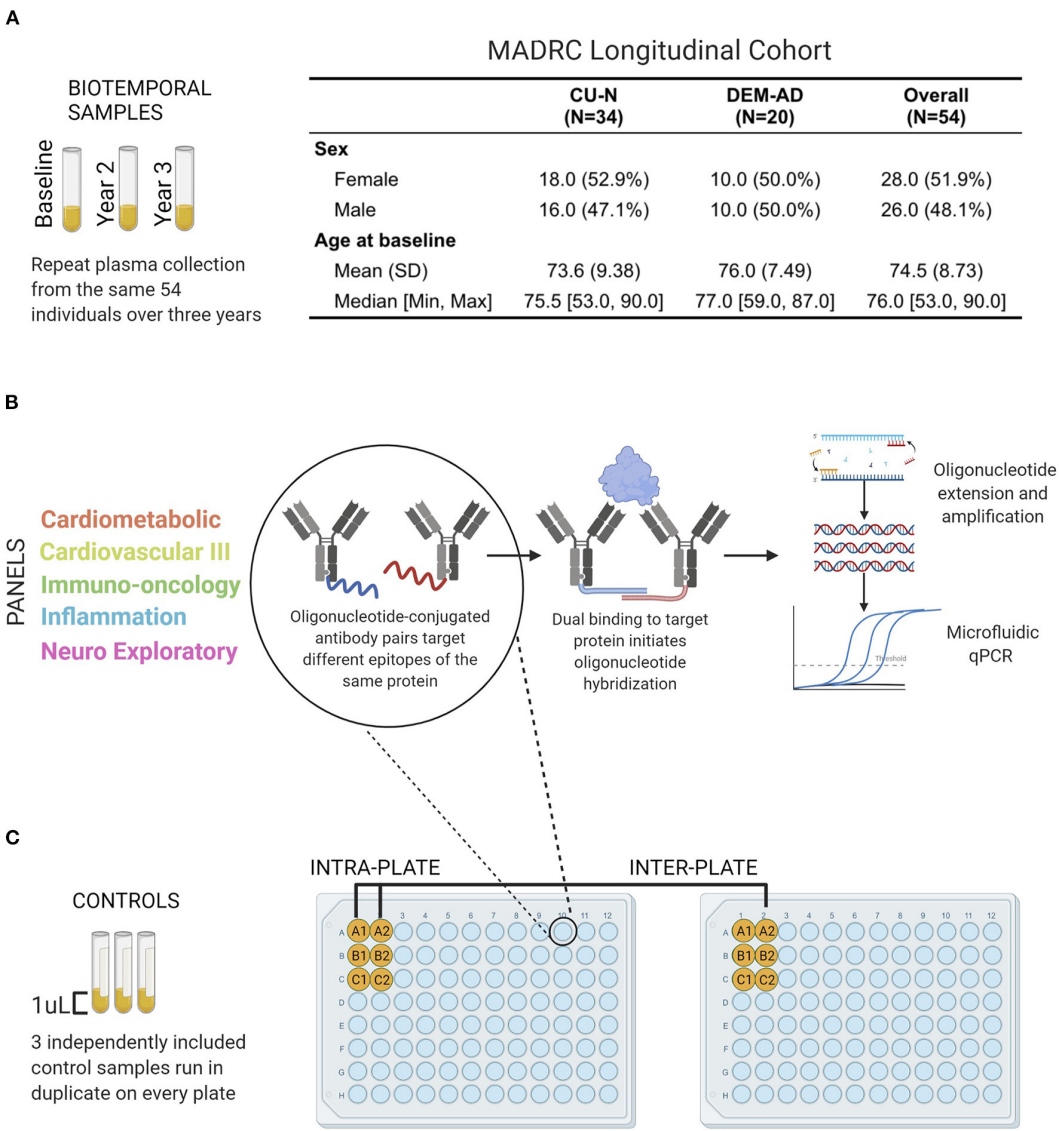
Performance evaluation schematic and Olink Proximity Extension Assay technology overview. **(A)** Plasma samples from 54 individuals of the Massachusetts Alzheimer's Disease Research Center Longitudinal cohort were analyzed by Olink PEA technology **(B)**. Olink PEA technology uses oligonucleotide-conjugated antibody pairs for targeting different epitopes of the same protein. Only when antibody pairs bind the same protein are the oligonucleotides close enough to hybridize. Annealed sequences are then extended, amplified, and measured by microfluidic qPCR. **(C)** Three control samples were run in duplicate on all plates and panels, allowing independent assessment of intra- and inter-plate assay precision.

Subjects were selected from the Massachusetts Alzheimer's Disease Research Center (MADRC) longitudinal cohort who were classified as AD (*n* = 20), or cognitively unimpaired (CU-N; *n* = 34) in standardized diagnostic consensus conferences (Figure 1A). Data available for diagnostic decision included at least one of detailed post-mortem neuropathology (*n* = 6), Amyloid β 11C-PiB-PET and ^18^F-flortaucipir PET imaging (*n* = 27), cerebrospinal fluid biomarkers (*n* = 7), structural MRI (*n* = 5), plasma tau levels (*n* = 2). For 7 subjects, the decision was based on longitudinal cognitive testing and basic clinical data only. Informed consent was obtained from all participants and collection and analysis of plasma samples from these individuals was approved by the Massachusetts General Hospital Institutional Review Board under protocols 2006P002104 and 1999P003693. Plasma was collected once per year for 3 years, a total of three time points per subject. Fourteen individuals had one interval between samples that was greater than 1.25 years but less than 2 years. Ten individuals had an interval of 2 years between sample collection. The median intervals between samples used in this study were 421 days for the interval between Visit 1 and Visit 2, and 428 days for the interval between Visit 2 and 3.

Sample collection was performed according to a consistent Standard Operating Procedure throughout the study, with samples proceeding from blood collection to aliquot freezing within 4 h. Blood was collected into EDTA collection tubes, inverted 10 times, and centrifuged at 2000 x g for 10 min at room temperature. After centrifugation, plasma was aliquoted into polypropylene tubes in 0.5 mL volumes and frozen at −80°C.

### Olink Analysis

Codified samples were sent to Olink Proteomics (Watertown, MA) for dilution and assays across five multi-analyte panels. Multiple investigators selected the panels that contained the highest number proteins of interest to AD biomarker research: Olink Target 96 Cardiometabolic (v.3602), Olink Target 96 Cardiovascular III (v.6112), Olink Target 96 Immuno-oncology (v.3103), Olink Target 96 Inflammation (v.3021), and Olink Target 96 Neuro Exploratory (v.3901). Olink's Proximity Extension Assay (PEA) technology uses antibody pairs conjugated to unique oligonucleotides and is quantified via PCR. When both antibodies of a pair bind the target protein simultaneously, their respective conjugated oligonucleotides are brought into proximity, facilitating hybridization. The oligonucleotide sequence is then extended by DNA polymerase, amplified, and measured by qPCR to determine the sample's initial protein abundance (12). Raw analyte expression values after PCR underwent multiple rounds of transformation by Olink, including a log2 transformation, and were returned as normalized protein expression (NPX) values (13). NPX values are not absolute quantifications, but an indication of relative concentration of each analyte. In total of 414 unique analytes were measured across the five protein panels, with each panel requiring only one microliter (μL) of plasma per sample.

### Data Analysis

Missing values per protein were counted across all samples, with those proteins with >20% missing values excluded from further analysis. Out of 460 total proteins quantified, 45 had >20 percent missing values and were excluded. Beyond Olink's internal controls, three technical control samples were included in duplicate on every plate to evaluate technical precision and reproducibility. Percent coefficient of variation (CV, variability between measurements of duplicate samples expressed as a percentage of mean abundance) was used as the measurement of intra- and inter-plate precision. CVs were calculated as follows; [(standard deviation / mean) ^*^ 100]. For intra-plate CVs, a CV was calculated for each same-plate duplicate sample pair, then averaged across all duplicate pairs. For inter-plate CV, the mean NPX value from each same-plate duplicate sample pair was calculated, and CV calculated across all plates using these mean values. Acceptable technical performance criteria were defined as CV <15%, with the CV threshold relaxed to 20% for biotemporal stability. Thirty six analytes are represented on more than one panel (for example, the ADA protein is measured on both the Immuno-Oncology and Inflammation panels), allowing additional calculation of multi-panel measurement correlations of the same analyte.

Olink normalization methods minimize the CVs across their in-house control samples, and the log2 transformation results in compression of these values. We therefore used a second approach to quantify the different types of variation inherent in each experiment, applying mixed effect ANOVA models and variance component analysis (VCA) to assess the proportion of variance introduced through potential technical and biological sources. For the technical variability experiments, the proportion of variance for each of three contributing variables (intraplate-, interplate-, and biological- variability) was computed as the ratio of mean square error for each variable vs. the sum of mean squares across all variables. For the analysis of biotemporal variation, where class size was strongly imbalanced, VCA was performed on these samples (using mixed-effect models via the *VCA* package in R) to determine the proportion of variance arising from diagnostic group, inter-individual differences, and biotemporal differences for each individual.

Finally, when taking measurements of multiple proteins simultaneously, consideration of initial sample size is critical. To demonstrate optimal sample size, power calculations were performed using baseline samples from all subjects. A two-sample two-tailed *t*-test power calculation was conducted using the base R power.t.test function. This was used to plot a power curve and exemplify sample size requirements for three analytes with markedly different effect sizes and inter-sample variability. The significance levels used equated to a Bonferroni corrected p-value for a one-protein experiment (0.05), 100-protein experiment (0.0005), and a 450-protein experiment (0.0001). All analyses were performed in R using the Table 1, tidyverse, and VCA packages (14).

## Results

### Experimental Design and Quality Control

**Figure 2:**
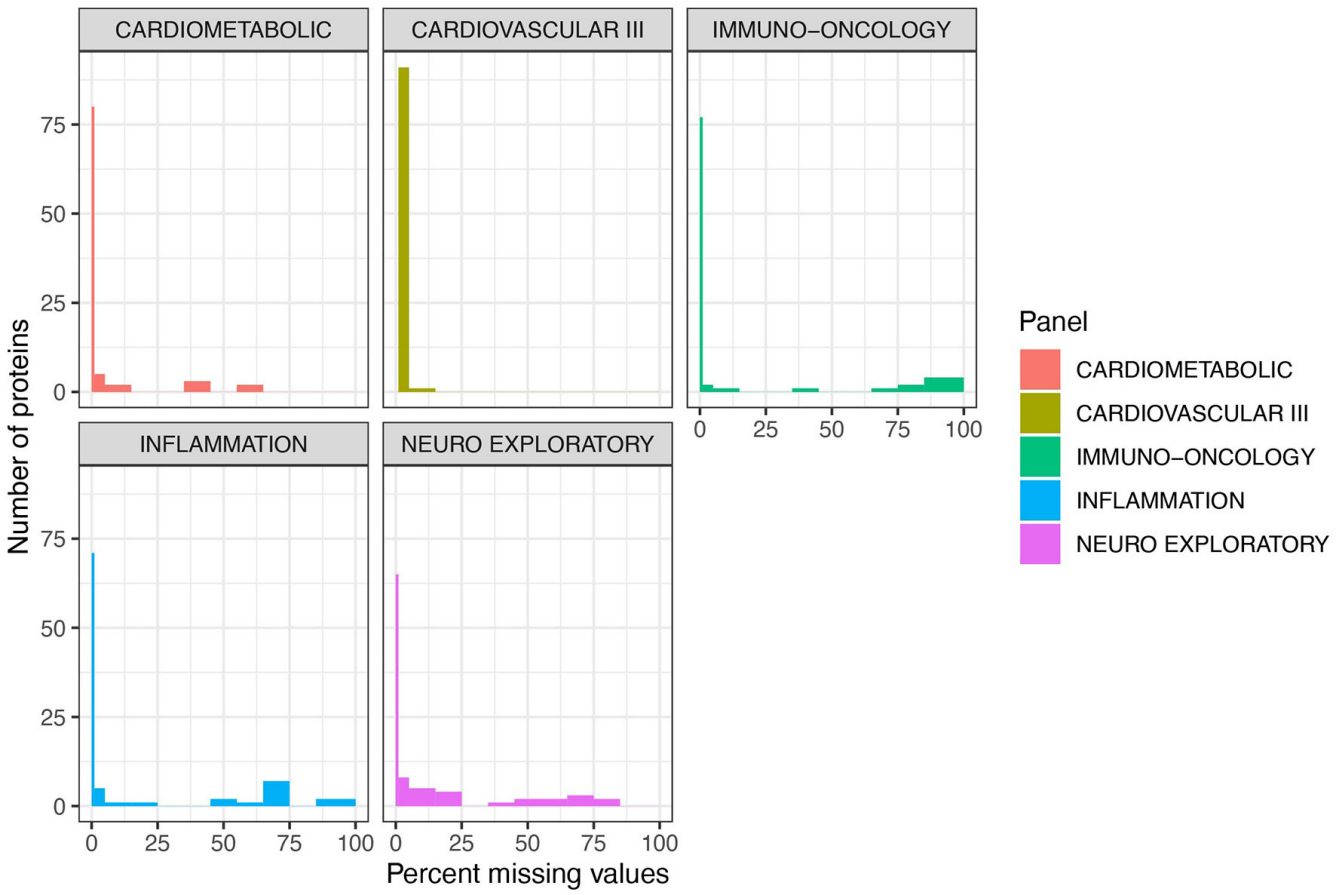
Cardiovascular III and Cardiometabolic panels demonstrate the lowest percentage of missing values per number of proteins. Five of Olink's multi-analyte panels [Cardiometabolic (v.3602), Cardiovascular III (v.6112), Immuno-oncology (v.3103), Inflammation (v.3021), and Neuro Exploratory (v.3901)] containing AD-relevant biomarkers were used to quantify 414 unique proteins. Across all five panels, 189 proteins had zero missing values. These proteins are shown with the thin line at 0 on these plots. Note that 1 sample failed on the Cardiovascular III panel, and thus there are zero proteins with zero missing values. Cardiometabolic and Cardiovascular III panels demonstrated the highest performance overall, with a very low number of proteins with a majority of missing values. Across all 5 panels, 45 proteins had over 20% missing values and were excluded from further analysis.

Fifty four individuals (CU-N, *n* = 34; AD, *n* = 20) with three approximately-yearly plasma samples available were selected from the Massachusetts Alzheimer's Disease Research Center Longitudinal cohort (Figure 1A). These samples underwent proteomic analysis across five Olink multi-analyte panels (Figure 1B). An additional three control samples (from three individuals) were run in duplicate across all plates and panels, to allow for independent assay stability assessments (Figure 1C). In total of 414 unique proteins were quantified across these five panels, with 189 proteins having zero missing values. The Cardiometabolic and Cardiovascular III panels had two and zero proteins with >50% missing values respectively, while the other three panels contained between 8 and 12 proteins with a majority of values missing (Figure 2, Supplementary Table S1). Proteins with over 20% missing values were excluded from further analysis, leaving a total of 415 proteins across five panels for assessment of technical performance. Due to the presence of the same protein on multiple panels, these 415 measurements corresponded to 377 unique proteins.

### Technical Precision and Variability

**Figure 3:**
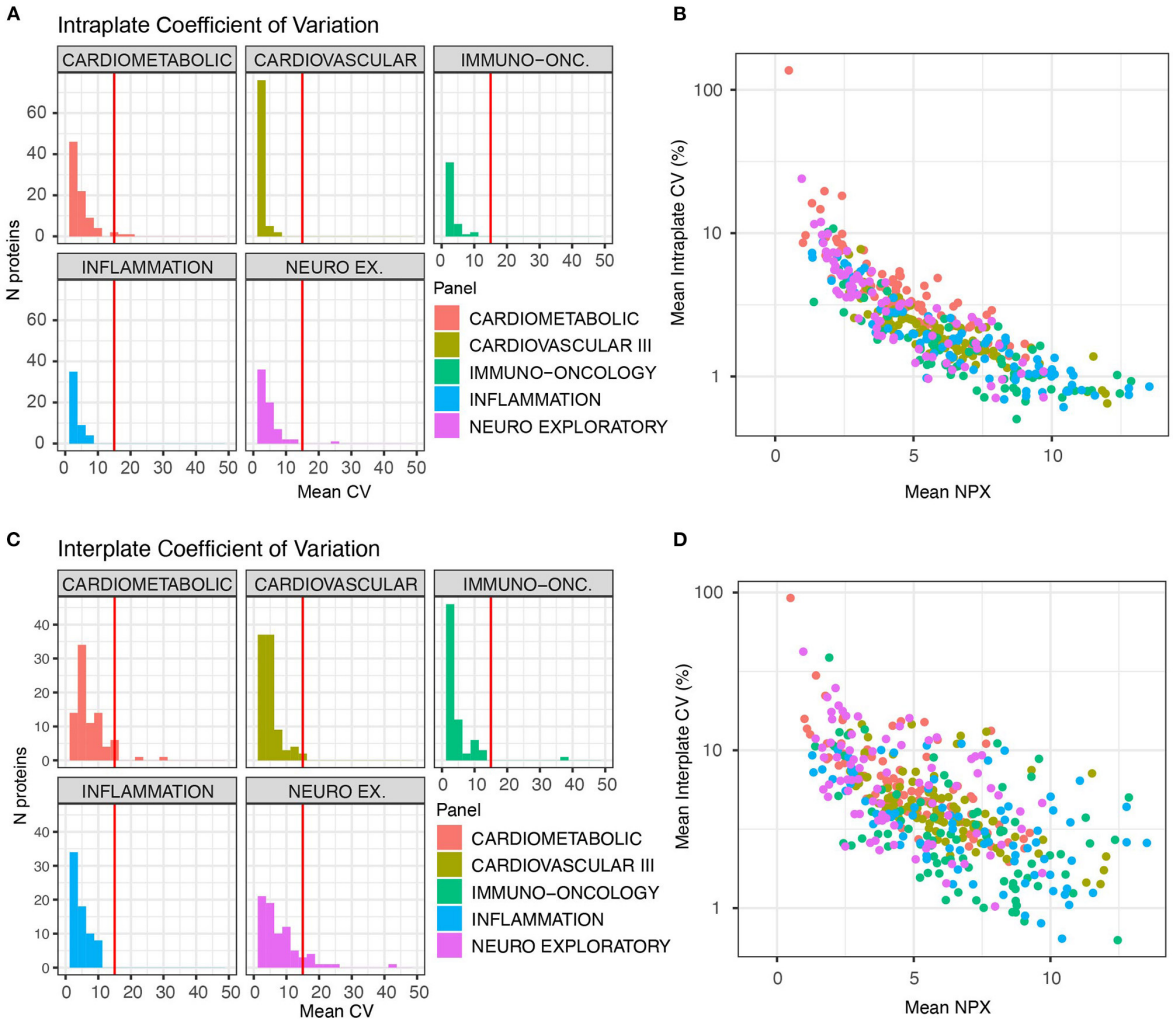
Proteins measured on Olink panels consistently demonstrate low CVs. Three control samples were run in duplicate on each plate across all panels for independent evaluation of assay precision and replicability. Coefficient of variation (CV) calculations were used to assess measurement reliability. The threshold of acceptable CV (15%) is marked with a vertical red line. **(A)** Intra-plate CVs for duplicates on the same plates. **(B)** Intraplate CV is strongly related to absolute NPX value. **(C)** Inter-plate CVs are marginally higher than intra-plate CVs. **(D)** As with intra-plate measurements, inter-plate CVs show a strong relationship to NPX value.

**Figure 4:**
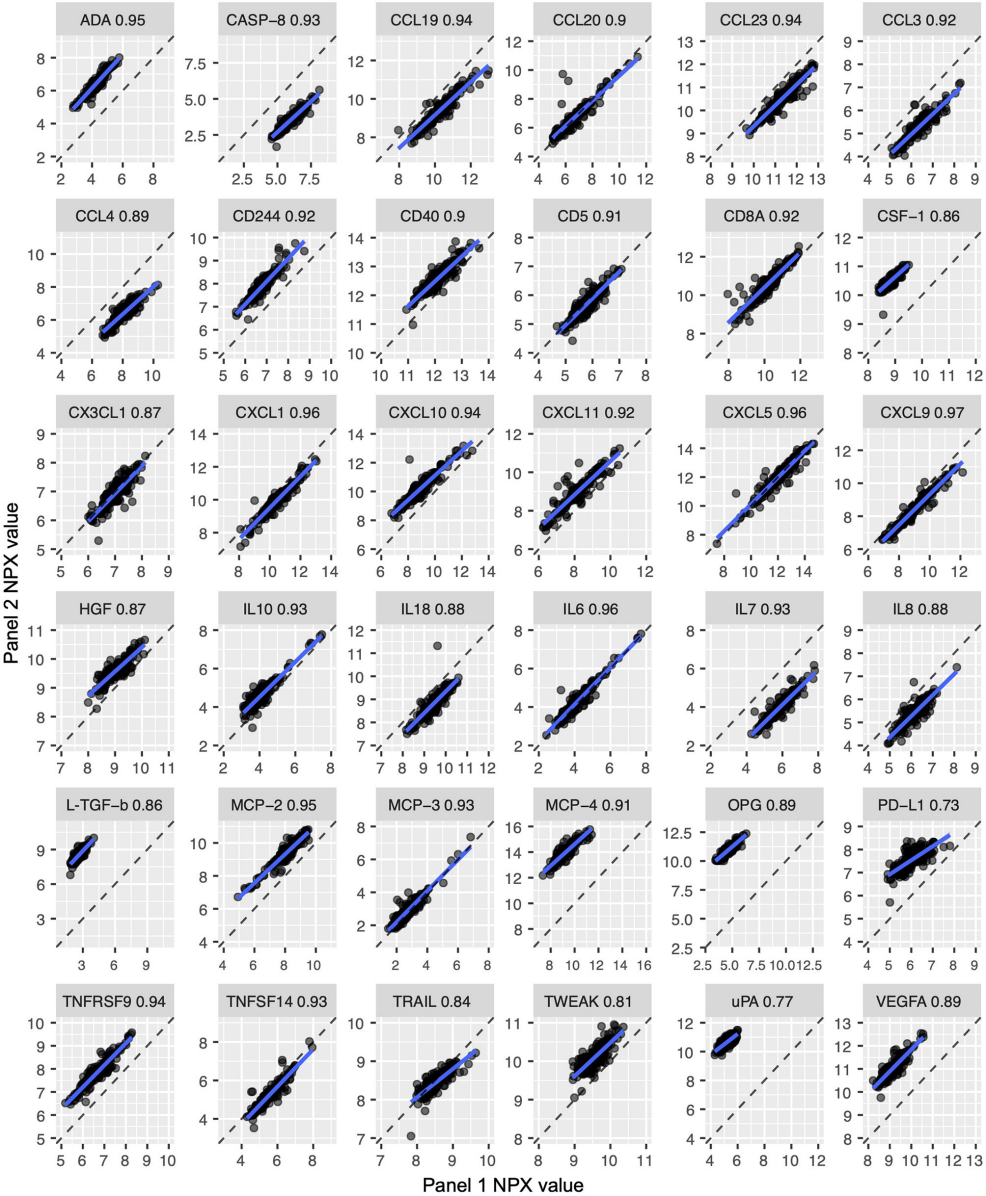
Proteins quantified on two different panels were highly correlated. Thirty six proteins were quantified on more than one panel (for example, the ADA protein is measured on both the Immuno-Oncology and Inflammation panels), which allowed analysis of inter-panel measurement precision. Each plot shows an individual analyte's measurements across two panels. The dashed line represents a perfect correlation of absolute values. While correlations are high (0.73–0.97), absolute values are not equivalent between panels.

The three technical control samples present in duplicate on every plate and panel were used for calculation of intra-plate (duplicate samples on the same plate) and inter-plate precision (duplicate samples on different plates) analyses. Intra-plate CVs were excellent, with only five measured analytes (CD59, FUT8, GNLY, ITGAM and FAP) displaying CVs >15% (Figure 3A, Supplementary Table S2). CVs were strongly related to analyte abundance, with lower NPX values resulting in higher CVs (Figure 3B). As expected, inter-plate CV distributions were marginally worse but still met performance thresholds, with 19 out of 415 measured proteins displaying CVs over 15% (Figure 3C, Supplementary Table S2). The majority of these analytes were on the Neuro Exploratory plex. As we observed with the intra-plate reliability, CVs were generally higher for analytes with lower NPX values (Figure 3D). Thirty six proteins were present on more than one panel that we tested, enabling us to assess correlation of values obtained from the same protein on different panels. Correlations were generally high, ranging from 0.73 (PD-L1) to 0.97 (CXCL9, Figure 4). However, as a result of variable dilution factors between plexes, NPX values were not in perfect agreement (Figure 4, dashed line shows perfect correlation of absolute values) for most proteins, suggesting that comparison between panels is only possible through relative changes and not NPX values.

**Figure 5:**
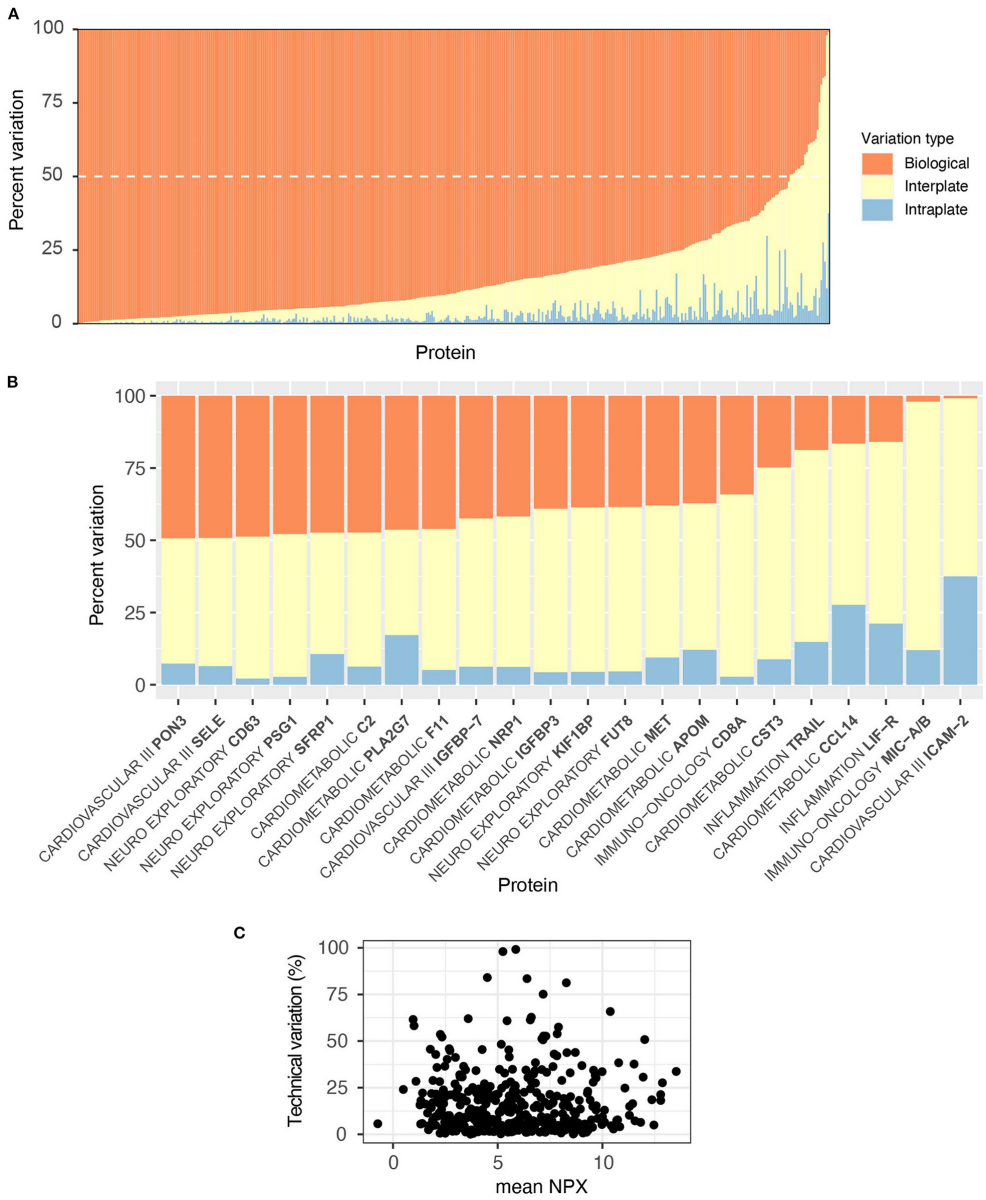
Olink is technically robust; individual differences are the largest source of variability. The proportion of technical (intra- and inter-plate) vs. biological sources of variance per protein was evaluated with an ANOVA on our independently included control samples. Variability arising from each source was expressed as a proportion of the sum of Mean Squares for that protein. **(A)** For all but 24 proteins, sample variability was primarily due to individual biological differences (orange bars), the dashed white line shows the 50% cut-off. **(B)** These 24 proteins, listed alongside their corresponding panel, were subject to a higher proportion of technical variability (>50%), shown in yellow and blue. **(C)** There was no relationship between proportion of technical variations (intra- plus inter-) and absolute NPX value.

As NPX values are log2 transformed, we were concerned that the CV statistic may not fully capture the technical variability in the data. We therefore also performed an ANOVA on data from the control samples, where we expressed the variation arising from intra-, inter-, and biological (between-individual) sources as a proportion of total variation for each protein. For all but 24 proteins (Figure 5B), individual differences among the three subjects account for the majority of the variability in the data (Figure 5A, Supplementary Table S3). Individual analytes are plotted by panel for inspection on Supplementary Figure S1. Unlike with CV, the proportion of technical variation was not related to absolute NPX value (Figure 5C). Comparison between the two analyses for intraplate variation showed a low Spearman correlation of 0.28, but concordance in identifying high performing analytes was high. In total of 312 proteins were identified as high performance by both analyses (intraplate CV below 5% and intraplate variation less than 5% of total), with 17 proteins identified as low performance by both analyses. Eighty six proteins were discordant between the two analyses. For interplate variation, Spearman correlation was slightly higher at 0.36, but concordance was lower. In total of 203 proteins were identified as high performance by both analyses (interplate CV below 5% and interplate variation below 20% of total), with 47 identified as low by both.

### Biological Variability

**Figure 6:**
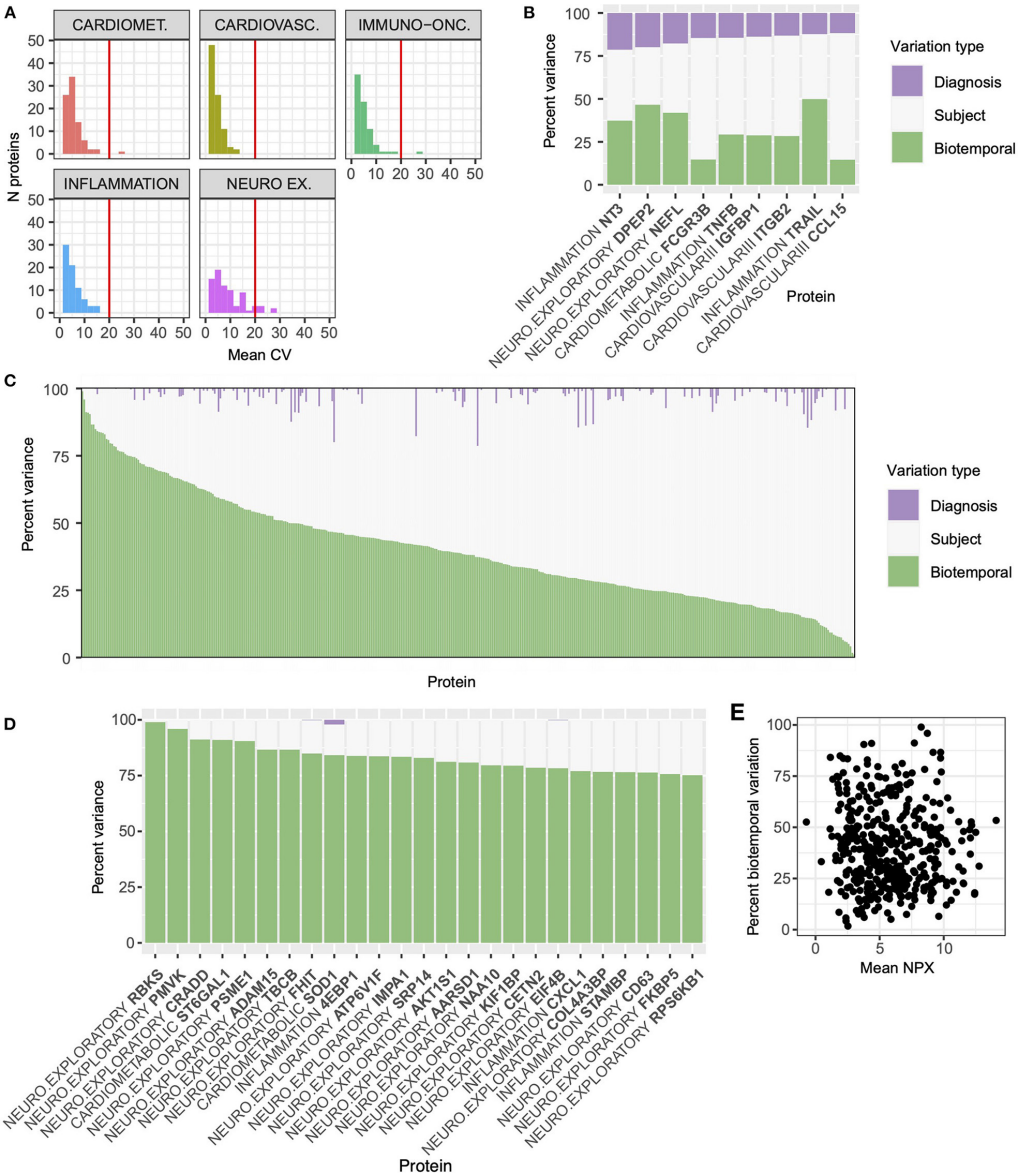
Inter-individual, not biotemporal, differences account for the majority of variance in most proteins. For the purpose of evaluating biotemporal analyte stability, intra-individual CV was calculated using 3 yearly plasma samples from the cognitively unimpaired subgroup (*n* = 34). **(A)** All but nine proteins met the 20 % threshold for acceptable biotemporal stability marked with the red vertical line. The Neuro-exploratory panel had the highest number of proteins whose biotemporal CV was above the acceptable threshold. **(B)** A VCA analysis was performed to assess the proportion of variability for each protein arising from diagnostic group (purple bars), Inter-individual (white), and intra-individual (biotemporal, green) sources. Nine proteins that have greater than 10% variation due to diagnostic group are highlighted. These nine proteins include the well-established blood biomarker NfL (NEFL). **(C)** For the majority of proteins (*n* = 320), inter-individual variation (white) was the largest source of variability. **(D)** Ninety three proteins demonstrated a high degree of biotemporal instability (green). Highlighted are proteins with biotemporal variance >75%. **(E)** Unlike inter- and intra-plate CVs, biotemporal variation component had no direct relationship with absolute NPX value across all proteins.

For a marker to be useful over time in longitudinal studies, including clinical trials, levels of the protein should be predictably stable in healthy individuals and not fluctuate much in response to common day-to-day factors such as diet, sleep, or diurnal changes. As the progression of AD occurs over long timescales, annual samples were selected that spanned a period of 3 years from baseline. A long-term biotemporal CV was calculated using the samples from each cognitively unimpaired individual. Only cognitively unimpaired samples were used for this calculation, as cognitive state was stable in these individuals. As observed with previous technical measures, most biotemporal CVs were acceptably low, with all but 9 proteins exhibiting a mean CV of less than 20 % (Figure 6A). In addition, all the analytes with high biotemporal variability had mean NPX values below 3.5, suggesting that instability of values may be a reflection of the previously observed relationship between high CV and low NPX values as opposed to a genuine reflection of biotemporal stability.

Due to the relationship observed between CV and NPX value, we also performed variance component analysis (VCA) on the serial samples from the cognitively unimpaired and AD samples and calculated the proportion of variance introduced by diagnosis, inter-individual differences, and intra-individual biotemporal differences/technical error. Only 9 proteins had >10% of their variance explained by diagnosis, including the well-established blood biomarker neurofilament-light (NfL, Figure 6B NEFL, Supplementary Table S4). NfL exhibited the third largest contribution from diagnostic contrast of any protein, with diagnostic group variation accounting for 17.8% of the total, and with biotemporal sources accounting for 41.9%. For 112 proteins, biotemporal differences accounted for >50% of the total variation, and for the other 320 proteins the majority of the variation is inter-individual (Figure 6C, Supplementary Table S4). Proteins with greater than 75% of variation arising from biotemporal instability are shown in Figure 6D. All proteins can be visualized by panel in Supplementary Figure S3. YKL40 showed a large amount of between subject variability (78.6%), with only 3% of total variability arising from diagnosis. Across all proteins there was no relationship between biotemporal variance component and absolute NPX value (Figure 6E), suggesting that a VCA may be a more appropriate method for assessing variability than a simple CV calculation. In Supplementary Figure S2 we show the trajectories for each individual sample of three proteins of interest to AD pathology. SMOC1 had the lowest biotemporal stability (mean CV = 3.04%, Supplementary Figure S2, Supplementary Table S2) and there is less than a 2-fold change in range between the maximum and minimum values. With specific relevance to established biomarkers in use for AD/ADRD research, neurofilament light (NfL) has a biotemporal CV of 5.1% and a broad range in maximum and minimum values (Supplementary Figure S2), while YKL40 (CHI3L1) has a biotemporal CV of 7.26%, with a similar broad range of values (Supplementary Figure S2).

**Figure 7:**
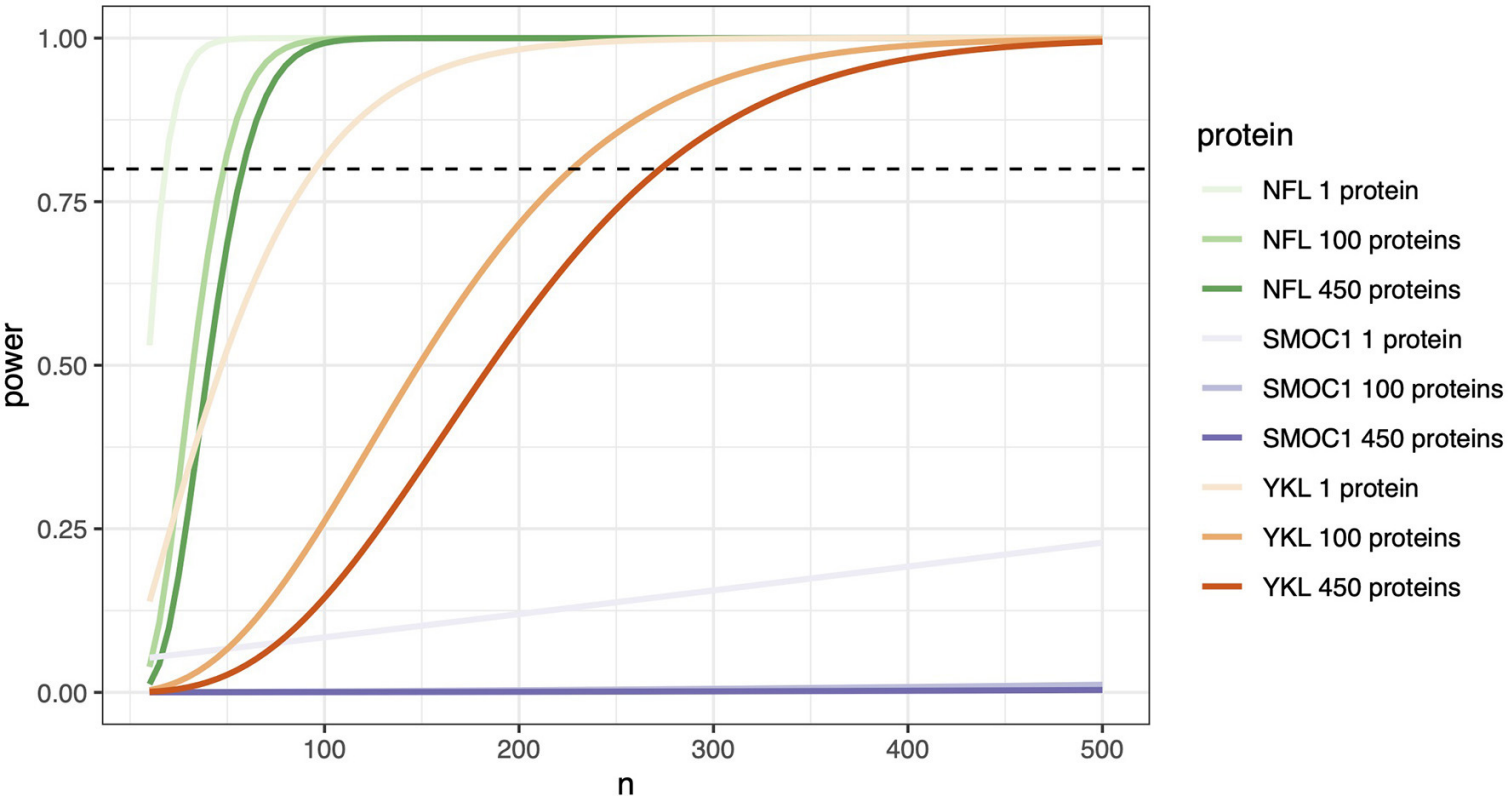
Protein effect size determines the number of samples needed. Stringent significance thresholds are necessary when measuring multiple proteins in parallel. Protein effect size becomes a significant determinant of the number of samples required to achieve confidence in any observed differences. A two-sample *t-*test power calculation on each individual's baseline data (*n* = 34 cognitively unimpaired and *n* = 20 AD) was used to simulate experimental power. Number of samples required in experiments measuring a single protein, 100 proteins, and 450 proteins were calculated. Significance values used corresponded to Bonferroni corrected *p*-values, 0.05 for a 1 protein experiment, 0.0005 for 100 proteins, and 0.0001 for 450 proteins. The power threshold of 80 % is represented here with the horizontal dashed line. In this model, proteins with significant effect sizes, such as NfL, can achieve 80% power in a single protein experiment with close to 30 samples per group, increasing only to 100 samples in an experiment measuring 450 proteins. In contrast, a protein with a moderate effect size such as MCP-1 requires an *n* =170 when measured alone, *n* = 400 if measuring *n* = 100 proteins, and just under 500 in an experiment of 450 proteins. SMOC1, even with 500 samples, fails to reach 80% power.

To explore how these distributions impact the power of each marker to detect a change between AD and controls, we took the baseline data from each individual (*n* = 54) and used this information to simulate experimental power using a simple two-sample *t*-test power calculation. To show the effect that multiple testing correction of *p*-values has on experimental power, we used significance levels that equated to a Bonferroni corrected *p*-value for a one-protein experiment (0.05), 100-protein experiment (0.0005), and a 450-protein experiment (0.0001). As expected for an established biomarker, NfL required approximately 20 samples per group to achieve 80% power in a single protein experiment (Figure 7). When the number of proteins increases to 450, approximately 60 samples per group are required. YKL40 has a delta similar to that of NfL (0.45 against NfL's 0.51, Supplementary Table S5), but a larger per group standard deviation, making the effect of multiple-testing correction much more severe. Approximately 95 samples per group are required in a single protein experiment to achieve 80% power, but in a 450-protein experiment, more than 270 samples per group are required. For SMOC1, despite having a low standard deviation, the between group delta is so small that 80% power is not reached in a 500-sample experiment (Figure 7, Supplementary Table S5).

## Discussion

Olink proximity extension assays are a relatively new technology, which is increasingly being used to try to identify novel biomarkers for AD in both cerebrospinal fluid and plasma. To our knowledge, the best powered plasma experiment to date involved 415 control individuals and 428 individuals with staged AD and the quantification of 250 proteins to identify a number of potential biomarkers. In the large discovery cohort, they identified 49 proteins changing in at least one disease contrast, 6 of which (OSM, MMP-9, HAGH, CD200, AXIN1, uPA) replicated in an independent cohort (15). In our study, OSM and MMP9 were 2 of the 116 proteins that showed non-zero contributions to variation from diagnosis by VCA. A smaller cohort of Hong Kong Chinese (*n* = 180) found 429 differentially abundant proteins in AD plasma (out of 1,160 tested), which included AXIN1 and uPA (16). In a large study of protein quantitative trait loci and Olink quantified serum proteomes in 2,893 individuals, CD33 protein was causally linked to AD disease traits (17). While AXIN, uPA, OSM and MMP9 may be emerging as potential biomarker targets, their performance and specificity is not yet well understood across multiple populations and neurological diseases.

To enable future discovery experiments using Olink technology, we comprehensively evaluated the performance of these assays in a small plasma cohort. The precision of Olink PEA technology was evaluated through an experimental design that included technical control samples on five commercial protein panels for biomarker discovery. Olink is an attractive technology for biomarker analysis as it measures a large number of proteins using a very small volume of sample (1μL). Frequently, samples are measured in singlicate, which does not allow for careful assessment of assay technical performance. Here, repeat sample measurements were used to determine measurement precision within and between plates, between panels, and with repeat sampling over multiple long-term timepoints. In accordance with standard criteria for immunocapture-based assays, Olink technology proved to be technically robust, providing acceptable performance for inter-and intraplate precision (CV <15%) for the majority of proteins. Analytes measured on multiple panels had high measurement correlation, although absolute NPX values were not always in agreement. NPX values are not absolute quantifications, but are relative measures of analyte concentration. These differences between panels are therefore a result of the variable dilution factors of samples used on the different panels. Despite the variable dilutions between panels, all measurements from our samples fell within the quantifiable range for these multi-panel proteins. Finally, CVs for long-term biotemporal stability in healthy controls were also very low.

Across all three contexts (intra-plate, inter-panel, and biotemporal), higher CVs were related to low NPX values, demonstrating that the closer that measurements are to the lower limit of detection (Supplementary Table S1), the more variable they become. Out of concern that this technical relationship and the log2 transformation of NPX values was not appropriately describing variability in these assays, we used additional approaches to model the impact of different sources of variation. An ANOVA showed that between-subject differences were the largest source of variation for the majority of analytes across duplicate measures of the same samples, suggesting that technical variability was acceptable for all but 24 proteins. Unlike with the CVs, where we observed a clear relationship between CV and absolute NPX value, there was no relationship between NPX magnitude and the proportion of technical variation measured by ANOVA. Although concordance between these two approaches in identifying high and low performing proteins was generally good, the comparison of these approaches suggests that CVs may not fully describe the variation inherent in these assays, and different approaches should be employed. In our analyses (VCA and ANOVA) of biotemporal and diagnostic sources of variation, we showed that while 93 proteins exhibited a large amount of biotemporal instability, most proteins exhibited much larger inter-individual differences. Again, there was no relationship between variance component due to biotemporal differences and absolute NPX value. The proteins that exhibited relatively higher biotemporal variability included Complement proteins CA1 and CA4, superoxide dismutase 1 (SOD1), and MMP9.

Large biotemporal variability within individuals may arise from a number of factors including circadian rhythms, environmental stressors, sleep, age, diet, disease processes, and many more known or unknown factors. These factors may complicate the use of such a protein as a biomarker in clinical trials, requiring specific test conditions such as fasting or carefully timed blood draws. In cross-sectional studies a protein with high biotemporal instability would require much larger sample sizes to overcome the day-to-day variability, or averaging of values obtained over multiple blood draws. It is therefore critical to examine the behavior of an analyte over relevant time periods. Fortunately, for the majority of the proteins quantified on these panels, there was greater inter-individual variation than intra-individual, suggesting that the majority of proteins on these five panels may be viable biomarkers for other disease conditions. However, in the particular context of AD versus controls studied here, only a small number of proteins varied in our diagnostic contrast of cognitively unimpaired to AD. These proteins included the established blood biomarker of neurodegeneration, NfL (18, 19). Two proteins had a greater proportion of variance arising from diagnosis than NfL; Neurotrophin-3 (NT3) and Dipeptidase-2 (DPEP2). NT3 is a neurotrophic factor that binds to TrkC receptors (20), may decrease with age in post-mortem hippocampus (21, 22), and has been shown to be increased in the CSF of elderly patients with Major Depressive Disorder (23), a key comorbidity of AD. In studies of microglial activation in aged mice, DPEP2 expression was coregulated with a biomarker of glial activation, YKL-40 (CHI3L3), and upregulated in the hippocampus after cerebral injections of cytokine cocktails (24), but to our knowledge has not been studied directly in AD.

A key consideration when multiplexing or performing unbiased discovery experiments on multi-analyte panels is the need for stringent statistical significance thresholds and replicate samples. This has a particularly strong effect on proteins with medium to small differences between diagnostic groups or with substantial within group standard deviations. To visualize this for future experiments using this technology, we plotted a power curve to model the influence of protein effect size on sample size requirements. A two-sample *t*-test calculation was performed using significance values that corresponded to Bonferroni corrected *p*-values. To model these effects we used Bonferroni correction, which only accounts for the number of tests performed, and is thus generalizable across different forms of experiment and different multiplexes. Alternative approaches which may be less stringent include Benjamini-Hochberg correction, which accounts for the *p*-value distribution, and thus may have a more moderate effect on *p*-value adjustment on a multiplex panel containing a number of proteins strongly related to Alzheimer's Disease.

With regard to experimental power in a 450-protein experiment, a higher effect size protein such as NfL required fewer than 50 samples per group to achieve confidence in observed significant differences. In contrast, the moderate effect size MCP-1 required almost 500 samples per group when 450 proteins are measured, decreasing to *n* = 170 if MCP-1 is measured alone. It is therefore important to carefully consider the number of proteins that are analyzed in multiplex experiments such as these and the number of samples available. Where sample numbers >500 are available, a hypothesis free approach that assesses hundreds of proteins across multiple panels may be taken. If only a small number of samples are available, then a more targeted approach should be considered. In the latter situation, it may be possible to formally develop a two-step analytical strategy, starting with a targeted à priori hypothesis analyzing one or two hypothesis-driven proteins, followed by a discovery analysis of all remaining proteins on the panel. This underpowered discovery analysis may be used to generate a hypothesis for further targeted testing in a replication cohort. As a resource for the community in planning future studies in AD using the Olink technology, we have provided effect-size and standard deviations, as well as pre-computed required sample sizes assuming an 80% power based on our data (Supplementary Table S5).

Olink is not an unbiased proteomic technology, but relies on the use of antibody pairs. As with all antibody-based technologies, the detection of proteins relies on the interaction of the antibody with the specific tertiary structure of the protein. This has the advantage that relative changes may be compared across multiple studies, as the antibody pair combinations will presumably measure the same proteoform species in all studies they are applied to. The use of antibodies allows PEA technology to probe further into the low abundance proteome than most standard current mass-spectrometry workflows. Compared to unbiased technologies such as liquid chromatography mass-spectrometry (2), missing data is very low, increasing the ability to easily compare data across studies. Previous studies have compared protein-level quantifications across PEA technology and two forms of MS, and found strong correlation of quantifications between technologies (25, 26). Depending on the goal of the project however, this specificity of quantified species may also be a limitation of these technologies. Unlike mass-spectrometry, which can be used to flexibly identify and quantify post-translational modifications such as phosphorylation, and the presence of novel protease-cleaved fragments, such as C-terminal TDP-43 (27), or specific processed peptides such as those from amyloid-β (28) or VGF (29), antibody pair technology does not have this flexibility.

In conclusion, Olink technology is technically reliable for the majority of analytes, proving to be a practical method for measuring a large number of proteins simultaneously while requiring a tiny volume of sample. Variability between analytes was due primarily to individual biological differences, as opposed to technical imprecision. Careful consideration of sample size should be made when using this technology in highly multiplexed or discovery research.

## Data Availability Statement

The datasets presented in this study can be found in online repositories. The name of the repository and accession number can be found below: Figshare, https://figshare.com/, doi: 10.6084/m9.figshare.19382867.v1.

## Ethics Statement

The studies involving human participants were reviewed and approved by Massachusetts General Hospital Institutional Review Board. The patients/participants provided their written informed consent to participate in this study.

## Author Contributions

BC, BH, PK, and SA designed the experiment. BT executed the experiment. BC, RK, ZM, AC, and SD performed analysis of the data. BC and ZM produced manuscript figures and supplementary tables and wrote the manuscript. BH and SA provided funding. All authors reviewed the manuscript. All authors contributed to the article and approved the submitted version.

## Funding

This work was supported by the Challenger Foundation/Minehan-Corrigan Family and by NIH R01 AG039478, R01 AG062306, P30 AG062421, and R01 AG17917. BC was supported by the Bright Focus Foundation. The plasma samples were obtained from the Harvard Biomarkers Study (HBS), which is a collaborative initiative of Brigham and Women's Hospital and Massachusetts General Hospital, co-directed by Dr. Clemens Scherzer and BH. HBS is made possible by generous support from the Harvard NeuroDiscovery Center, with additional contributions from the Michael J Fox Foundation, NINDS U01NS082157, U01NS100603, and the Massachusetts Alzheimer's Disease Research Center NIA P50AG005134.

## Conflict of Interest

The authors declare that the research was conducted in the absence of any commercial or financial relationships that could be construed as a potential conflict of interest.

## Publisher's Note

All claims expressed in this article are solely those of the authors and do not necessarily represent those of their affiliated organizations, or those of the publisher, the editors and the reviewers. Any product that may be evaluated in this article, or claim that may be made by its manufacturer, is not guaranteed or endorsed by the publisher.

## Supplementary Material

Supplementary Figure S1 Proportion of variability arising from intra-plate (blue), inter-plate (yellow), and biological (orange), sources in the technical control samples, plotted per protein and panel.

Supplementary Figure S2 Fluctuations over time in AD-associated proteins. The trajectories over time of three analytes of interest is demonstrated by plotting NPX value across each timepoint per each individual. NPX values are log2 transformed during data processing. YKL40 has the greatest biotemporal instability (CV = 7.26%, Supplementary Table S2) of the three, with NPX value often changing more than 0.5 NPX between time points, and the largest range in values between subjects. NfL has a biotemporal CV of 5.1% (Supplementary Table S2). SMOC1 is the most stable over time, with the lowest biotemporal CV (3.04%, Supplementary Table S2).

Supplementary Figure S3 Proportion of variability in the biotemporal sample set arising from diagnostic group (purple), inter-individual (white) and intra-individual (biotemporal, green) sources, plotted per protein and panel.

Supplementary Table S1 Summary of the proportion of missing values (NA) per protein and panel. Final column shows the Limit of Detection (LOD) for each protein.

Supplementary Table S2 Summary of intra-, inter-plate, and biotemporal CVs of each protein per panel.

Supplementary Table S3 ANOVA outcome results. The total Mean Squares and the proportion of variance (Mean Square source/Sum of Mean Squares) arising from each source of variation is shown.

Supplementary Table S4 VCA outcome results. This table shows the proportion of variance arising from diagnostic group, inter-subject, and intra-subject (biotemporal) sources for each protein on each panel.

Supplementary Table S5 Power calculations. This table shows delta, standard deviation, and the required n per group for a 1 protein, 100 protein and 450 protein experiment to reach 80% power for each protein.

## References

[1] Shi L Baird AL Westwood S Hye A Dobson R Thambisetty M. A decade of blood biomarkers for alzheimer's disease research: an evolving field, improving study designs, and the challenge of replication. J Alzheimer's Dis. (2018) 62:1181–98. 10.3233/JAD-170531 29562526 PMC5870012

[2] Carlyle B Trombetta B Arnold S. Proteomic approaches for the discovery of biofluid biomarkers of neurodegenerative dementias. Proteomes. (2018) 6:32. 10.3390/proteomes6030032 30200280 PMC6161166

[3] Jack CR Holtzman DM. Biomarker modeling of Alzheimer's disease. Neuron. (2013) 80:1347–58. 10.1016/j.neuron.2013.12.003 24360540 PMC3928967

[4] Peskind ER Riekse R Quinn JF Kaye J Clark CM Farlow MR. Safety and acceptability of the research lumbar puncture. Alzheimer Dis Assoc Disord. (2005) 19:220–5. 10.1097/01.wad.0000194014.43575.fd 16327349

[5] Zetterberg H. Cerebrospinal fluid biomarkers for Alzheimer's disease: current limitations and recent developments. Curr Opin Psychiatry. (2015) 28:402–9. 10.1097/YCO.0000000000000179 26147615

[6] Santiago JA Potashkin JA. The impact of disease comorbidities in Alzheimer's disease. Front Aging Neurosci. (2021) 13:631770. 10.3389/fnagi.2021.631770 33643025 PMC7906983

[7] Hall JR Wiechmann AR Johnson LA Edwards M Barber RC Winter AS. Biomarkers of vascular risk, systemic inflammation, and microvascular pathology and neuropsychiatric symptoms in Alzheimer's disease. J Alzheimer's Dis. (2013) 35:363–71. 10.3233/JAD-122359 23403534 PMC3631280

[8] Arnold SE Arvanitakis Z Macauley-Rambach SL Koenig AM Wang H-Y Ahima RS. Brain insulin resistance in type 2 diabetes and Alzheimer disease: concepts and conundrums. Nat Rev Neurol. (2018) 14:168–81. 10.1038/nrneurol.2017.185 29377010 PMC6098968

[9] Trombetta BA Carlyle BC Koenig AM Shaw LM Trojanowski JQ Wolk DA. The technical reliability and biotemporal stability of cerebrospinal fluid biomarkers for profiling multiple pathophysiologies in Alzheimer's disease. PLoS ONE. (2018) 13:e0193707. 10.1371/journal.pone.0193707 29505610 PMC5837100

[10] Wu D Dinh TL Bausk BP Walt DR. Long-term measurements of human inflammatory cytokines reveal complex baseline variations between individuals. Am J Pathol. (2017) 187:2620–6. 10.1016/j.ajpath.2017.08.007 28919109

[11] Lewczuk P Riederer P O'Bryant SE Verbeek MM Dubois B Visser PJ. Cerebrospinal fluid and blood biomarkers for neurodegenerative dementias: an update of the Consensus of the Task Force on Biological Markers in Psychiatry of the World Federation of Societies of Biological Psychiatry. World J Biol Psychiatry. (2017) 19:244–328. 10.1080/15622975.2017.1375556 29076399 PMC5916324

[12] Lundberg M Eriksson A Tran B Assarsson E Fredriksson S. Homogeneous antibody-based proximity extension assays provide sensitive and specific detection of low-abundant proteins in human blood. Nucleic Acids Res. (2011) 39:e102. 10.1093/nar/gkr424 21646338 PMC3159481

[13] Assarsson E Lundberg M Holmquist G Björkesten J Thorsen SB Ekman D. Homogenous 96-plex PEA immunoassay exhibiting high sensitivity, specificity, and excellent scalability. PLoS ONE. (2014) 9:e95192. 10.1371/journal.pone.0095192 24755770 PMC3995906

[14] R Foundation for Statistical Computing. R: A Language and Environment for Statistical Computing. Vienna: R Foundation for statistical computing 3.3.1. (2016).

[15] Whelan CD Mattsson N Nagle MW Vijayaraghavan S Hyde C Janelidze S. Multiplex proteomics identifies novel CSF and plasma biomarkers of early Alzheimer's disease. Acta Neuropathol Commun. (2019) 7:169. 10.1186/s40478-019-0795-2 31694701 PMC6836495

[16] Jiang Y Zhou X Ip FC Chan P Chen Y Lai NCH. Large-scale plasma proteomic profiling identifies a high-performance biomarker panel for Alzheimer's disease screening and staging. Alzheimer's Dement. (2022) 18:88–102. 10.1002/alz.12369 34032364 PMC9292367

[17] Png G Barysenka A Repetto L Navarro P Shen X Pietzner M. Mapping the serum proteome to neurological diseases using whole genome sequencing. Nat Commun. (2021) 12:7042. 10.1038/s41467-021-27387-1 34857772 PMC8640022

[18] Forgrave LM Ma M Best JR DeMarco ML. The diagnostic performance of neurofilament light chain in CSF and blood for Alzheimer's disease, frontotemporal dementia, and amyotrophic lateral sclerosis: a systematic review and meta-analysis. Alzheimer's Dement Diagnosis. Assess Dis Monit. (2019) 11:730. 10.1016/j.dadm.2019.08.009 31909174 PMC6939029

[19] Jin M Cao L Dai Y. Role of neurofilament light chain as a potential biomarker for alzheimer's disease: a correlative meta-analysis. Front Aging Neurosci. (2019) 11:254. 10.3389/fnagi.2019.00254 31572170 PMC6753203

[20] Lamballe F Klein R Barbacid M. trkC, a new member of the trk family of tyrosine protein kinases, is a receptor for neurotrophin-3. Cell. (1991) 66:967–79. 10.1016/0092-8674(91)90442-2 1653651

[21] Durany N Michel T Kurt J Cruz-Sánchez FF Cervós-Navarro J Riederer P. Brain-derived neurotrophic factor and neurotrophin-3 levels in Alzheimer's disease brains. Int J Dev Neurosci. (2000) 18:807–13. 10.1016/S0736-5748(00)00046-0 11154850

[22] Hock C Heese K Hulette C Rosenberg C Otten U. Region-specific neurotrophin imbalances in Alzheimer disease: decreased levels of brain-derived neurotrophic factor and increased levels of nerve growth factor in hippocampus and cortical areas. Arch Neurol. (2000) 57:846–51. 10.1001/archneur.57.6.846 10867782

[23] Hock C Heese K Müller-Spahn F Huber P Riesen W Nitsch RM. Increased cerebrospinal fluid levels of neurotrophin 3 (NT-3) in elderly patients with major depression. Mol Psychiatry. (2000) 5:510–3. 10.1038/sj.mp.4000743 11032384

[24] Lee DC Ruiz CR Lebson L Selenica M-LB Rizer J Rojiani R. Aging enhances classical activation but mitigates alternative activation in the CNS. Neurobiol Aging. (2013) 34:1610. 10.1016/j.neurobiolaging.2012.12.014 23481567 PMC3652232

[25] Petrera A von Toerne C Behler J Huth C Thorand B Hilgendorff A. Multiplatform approach for plasma proteomics: complementarity of olink proximity extension assay technology to mass spectrometry-based protein profiling. J Proteome Res. (2021) 20:751–62. 10.1021/acs.jproteome.0c00641 33253581

[26] Arrigo M Vodovar N Von Moos S Masson E Segerer S Cippà PE. High accuracy of proximity extension assay technology for the quantification of plasma brain natriuretic peptide. J Clin Lab Anal. (2018) 32:e22574. 10.1002/jcla.22574 29797353 PMC6817038

[27] Feneberg E Charles PD Finelli MJ Scott C Kessler BM Fischer R. Detection and quantification of novel C-terminal TDP-43 fragments in ALS-TDP. Brain Pathol. (2021) 31:e12923. 10.1111/bpa.12923 33300249 PMC8412074

[28] Braun GA Dear AJ Sanagavarapu K Zetterberg H Linse S. Amyloid-β peptide 37, 38 and 40 individually and cooperatively inhibit amyloid-β 42 aggregation. Chem Sci. (2022) 13:2423–39. 10.1039/D1SC02990H 35310497 PMC8864715

[29] Quinn JP Kandigian SE Trombetta BA Arnold SE Carlyle BC. VGF as a biomarker and therapeutic target in neurodegenerative and psychiatric diseases. Brain Commun. (2021) 3:fcab261. 10.1093/braincomms/fcab261 34778762 PMC8578498

